# Hidradenitis suppurativa: Basic considerations for its approach: A narrative review

**DOI:** 10.1016/j.amsu.2021.102679

**Published:** 2021-08-05

**Authors:** Felipe Anduquia-Garay, María Manuela Rodríguez-Gutiérrez, Ingrith Tatiana Poveda-Castillo, Pedro Luis Valdes-Moreno, Diego Alexander Agudelo-Rios, Juan Sebastián Benavides-Moreno, Ivan David Lozada-Martínez, Maria Paz Bolaño-Romero, Bernardo Borraez-Segura, Sabrina Rahman

**Affiliations:** a Future Surgeons Chapter, Colombian Surgery Association, Bogotá, Colombia; b School of Medicine, Universidad Tecnológica de Pereira, Pereira, Colombia; c Medical and Surgical Research Center, School of Medicine, Fundación Universitaria Autónoma de las Americas, Pereira, Colombia; d School of Medicine, Fundación Universitaria Juan N Corpas, Bogotá, Colombia; e School of Medicine, Universidad Cooperativa de Colombia, Santa Marta, Colombia; f School of Medicine, Fundacion Universitaria San Martin, Medellín, Colombia; g School of Medicine, Fundación Universitaria Sanitas, Bogotá, Colombia; h Medical and Surgical Research Center, University of Cartagena, Cartagena, Colombia; i Department of Public Health, Independent University-Bangladesh, Dhaka, Bangladesh

**Keywords:** Hidradenitis suppurativa, Diagnosis, Therapeutics, Skin disease, Surgery

## Abstract

Hidradenitis suppurativa is a chronic and debilitating skin disease, whose lesions can range from inflammatory nodules to abscesses and fistulas in the armpits, groin, perineum, inframammary region. Diagnosis can be confused with a large number of clinical pictures, and although studies on hidradenitis suppurativa are not so scarce in the literature, doctors are often unaware of this disease and therefore its diagnosis is often late. Pharmacological treatment ranges from retinoids to immunosuppression and radiation therapy, and surgical treatment ranges from incision and drainage to more complete excisions and laser therapies. Hidradenitis suppurativa is a disease seen and treated mainly by dermatologists and general surgeons, however, it is necessary for general practitioners to have basic knowledge about this entity, as they are the first line of care in the health system.

## Highlights

• Hidradenitis suppurativa is an entity of necessary recognition by the surgeon.
• It is a preventable and treatable condition with a good prognosis.
• This condition should be managed by a multidisciplinary team.

## 1 Introduction

Hidradenitis suppurativa (HS) is a chronic skin disease characterized by the presence of difficult-to-manage inflammatory nodules that can progress to abscesses and fistulas in areas such as armpits, groin, perineum, inframammary region, and other intertriginous areas [1]. A 2017 study showed a prevalence of HS of 0.1%, and an incidence more than twice in women, with a peak age between 30 and 39 years [2,3]. HS can be confused with a large number of diseases such as acne or insect bites; for this reason, its diagnosis is usually late. It is mistakenly confused with lesions of infectious origin; however, it has been described that this is secondary and not a cause of HS [4]. Studies have shown that the majority of HS patients visited a physician at least five times before receiving a diagnosis and the average time before diagnosis was around seven years [5,6]. If not treated in time, HS can lead to severe long-term sequelae such as genitourinary stenosis (in the case of affecting the genitourinary region) and an association with squamous cell carcinoma has even been described [7].

Although the exact cause is unknown, risk factors for the development of HS are obesity, smoking and other inflammatory disorders such acne, pyoderma gangrenosum, arthritis, diabetes mellitus, and inflammatory bowel disease [8]. Because a very high number of lesions can be located in the groin area, HS dramatically influences the sexual and emotional health of the patient [9]. Although its treatment and cure are difficult, there are different treatments depending on the severity of the disease for early stages, there is medical therapy such as antibiotics, retinoids, immunosuppressants, anti-inflammatories and radiotherapy, and combined therapies with oxygen therapy and laser, as well as surgical interventions for more severe stages of the disease [10]. Based on the above, and the importance of early diagnosis and effective symptomatic treatment while the definitive diagnosis is being established, the objective of this review is to present basic considerations in the approach to hidradenitis suppurativa, in order to serve as a guide for the general practitioner.

## 2 Causes

HS appears to be a follicular occlusion disease of unclear etiology. The causes of HS are probably multifactorial. Endogenous factors include genetic predisposition, aberrant immunity, and hormonal influences. The important exogenous factors in the pathogenesis of HS are smoking, obesity, bacterial infections, friction and probably socioeconomic factors [11]. Some of the factors described are:

- Genetic factors: Has been reported in approximately 35%–40% of patients, to date, several genetic loci that are associated with HS have been identified, but so far, no causative genes have been found. Mutations were identified in the genes of the γ-secretase, genes of the NOTCH pathway, PSENEN (presenilin-enhancing gamma-secretase subunit), PSEN1 (presenilin 1), NCSTN (nicastrin), when γ-secretase function is impaired in mice, follicular keratinization, follicular atrophy, epidermal cyst formation, absence of sebaceous glands, and epidermal hyperplasia occur, features that can also be found in HS [12,13].
- Aberrant immune response: The chronic inflammatory nature combined with the frequent absence of pathogenic bacteria is suspicious for the role of aberrant immunity in HS. This suspicion is reinforced by the reported co-occurrence with sterile arthritis and the presumed association and similarities with Crohn's disease. Several inflammatory cytokines are elevated in HS lesions, including IL-1β, TNF-α IL-10, CXCL9, interferon-γ-induced monokine, IL-11, chemoattractant of B lymphocytes and IL-17A [11,12].
- Hormonal Influences: A relationship between HS and hormones seems conceivable, since the onset of the disease occurs mainly after puberty and the prevalence gradually decreases after menopause. Studies of HS in children are associated with premature adrenochemia or precocious puberty, when androgens are dominant. As in acne, there are no increases in serum androgens in most HS19 patients and the effect is assumed to be caused by end-organ sensitivity [[11], [12], [13]].
- Smoking: Smoking is a factor that is strongly associated with HS. The patients who smoke tend to have a more serious illness than non-smokers. It has been demonstrated that nicotine promotes inflammatory reactions, such as causing mast cell degranulation and improving neutrophil survival and chemotaxis. Furthermore, in vitro models it has been shown that nicotine can induce epidermal hyperplasia and follicular plugging [11,14].
- Obesity: As the majority of HS patients (45-80%) are overweight (body mass index (BMI) > 25 kg/m2. Furthermore, obese patients (BMI> 30 kg/m2) have been shown to have a more severe disease presentation than normal weight patients. Several hypotheses are put forward why obesity may contribute to the development of HS. First, increased skin-to-skin contact increases mechanical friction, which may cause micro-tears of hair follicles in predisposed individuals. Second, overlapping skin folds can cause perspiration retention, which can cause irritation and maceration when combined with friction, leading to skin inflammation. Third, bacterial growth is favored by the often warm and humid microclimate present in the skin folds, leading to secondary infections of the inflamed skin. Fourth, obese patients have neither Elevated level of circulating pro-inflammatory cytokines are also elevated in the skin of HS patients [11,13].
- Bacterial infection: Over time it was thought that bacterial infection affected the development of HS. However, bacterial cultures of HS lesions are often sterile, assuming that bacterial colonization is a secondary event [11,12].
- External factors: The role of shaving, the use of chemical depilatories, deodorants and talc as causative factors of HS. Patients report that wearing tight clothing increases the number of inflammatory lesions and that wearing loose clothing decreases the symptoms of their HS, the role of mechanical friction is the main mechanism by which HS occurs in these patients [11].
- Socioeconomic status: Although there is a perception that HS occurs more frequently in patients from lower socioeconomic classes, there is limited evidence to support this hypothesis [11].

## 3 Clinical manifestations

HS is a chronic inflammatory disorder characterized by the presence of very painful nodules, abscesses and fistulas, frequently accompanied by chronic drainage. The lesions are located in fold areas where apocrine glands are present, such as the axillary, inguinal, perianal, inframammary and femoral regions [[15], [16], [17]].

The clinical presentation is highly variable in terms of the distribution of the lesions, the presence of complications such as scars restricted mobility of the extremities, strictures or fistulas in the anus and urethra due to chronic inflammation, lymphedema or scrotal elephantiasis, extracutaneous characteristics such as interstitial keratitis and associated constitutional, fever and general malaise. In addition, it is important to take into account that the presence of aggressive squamous cell carcinoma has been reported in areas with chronic scars, which worsens its prognosis [13,18].

Primary lesions are individual painful nodules, 0.5–2 cm in diameter that persist for weeks to months with a variable degree of inflammation. These lesions can be deep and patients complain of pain, although the only visible thing is redness with little swelling, despite the great discomfort, it is accompanied by burning, stinging, pain and itching, with or without hyperhidrosis. Secondary lesions develop as a result of the persistence of the process in one area. The subcutaneous coalescence of several neighboring cysts results in the formation of chronic interrelated sinuses. Drainage from these lesions may be serous, purulent, or bloody. Tertiary injuries occur as a result of aberrant scarring [12]. These lesions begin to appear after puberty, approximately between 20 and 30 years, the onset at an earlier age has been associated as an indicator of severity [19].

## 4 Diagnosis

Hidradenitis suppurativa has a clinical diagnosis and remains a diagnostic challenge. Often misdiagnosed, it can take 12 years or more from the onset of symptoms to diagnosis. Consensus diagnostic criteria require that individuals have typical lesions (painful nodules, abscesses, sinus tracts, bridging scars, or open comedones) at typical sites (armpits, groin, perineal region, perianal region, inframammary and intermammary or gluteal folds) and that the disease must be chronic and recurrent. The initial study may include measurement of the complete blood count, routine chemistry, and inflammatory markers. Other elements that should be obtained in the history include a family history of HS, initial onset of symptoms, and gastrointestinal symptoms given the association with Crohn's disease [13].

For patients with lesions at atypical sites for hidradenitis suppurativa, screening for conditions with defective immune function may include complete blood count, differential, and peripheral smear immunoglobulin (Ig) A, IgM, IgG, and IgE tests, total complement levels, HIV testing, serum protein electrophoresis, urine for Bence-Jones protein [19].

There are limitations for the determination of severity when there is existence of subclinical lesions not identifiable in the general physical examination. Since, at the time of clinical palpation, it has a low sensitivity when differentiating between inflammatory and non-inflammatory nodules and fistulas, so it is vitally important to request an ultrasound to find diffuse alteration of the dermal pattern, a dermal thickening, the presence of dermal pseudocysts, the thickening of the hair follicle and the detection of fluid collections and fistulous tracts, for which the SOS-HS **(**Table 1**)** [20].

**Table 1:** SOS-HS (clinical-sonographic scoring system) [20].

Stage	Description
I	A single fluid collection and dermal changes (presence of hypo- or anechoic pseudocystic nodules, thickening of hair follicles, alterations in dermal thickness), affecting one body area (e.g. axilla, groin, breast, buttock) (uni or bilateral). No presence of fistulous tracts.
II	2 to 4 fluid collections or a fistulous tract, with dermal changes affecting one or 2 body areas (uni or bilateral).
III	≥5 fluid collections or ≥2 fistulous tracts, with dermal changes or involvement of ≥3 body areas (uni or bilateral).

Assessment of disease severity is helpful in guiding treatment and is generally based on the Hurley staging system **(**Table 2**)** [20]. The Hurley system is relatively insensitive to change, so other instruments are used to measure the effectiveness of treatment. Patient-reported domains include pain, measured with a visual analog scale or numerical rating scale (0-10), and quality of life, measured with a dermatology-specific scale such as the DLQI (Dermatology life quality index), Skindex, Sartorius score, IHS-4 **(**Table 3**)** [12,21,22].

**Table 2:** Hurley classification [20].

Stage	Description of skin changes
I	Abscesses single or multiple, no fistulas and scars
II	Chronic abscesses with fistulas and scars, single or multiple
III	Disseminated changes in several location with abscesses, bridge-type scars and fistulas

**Table 3:** IHS-4 (International hidradenitis suppurativa severity score system) [21].

HIS-4	Points*
Number or nodules	x 1 +
Number of abscesses	x 2 +
Number of draining tunnels (fistulae/sinuses)	x 4
Mild HS	≤3 points
Moderate HS	4-10 points
Severe HS	≥11 points

*The proposed new International HS4 (IHS4) is: IHS4 (points) = (number of nodules multiplied by 1) + (number of abscesses multiplied by 2) + [number of draining tunnels (fistulae/sinuses) multiplied by 4]. A score of 3 or less signifies mild, a score of 4–10 signifies moderate and a score of 11 or higher signifies severe HS.

## 5 Pharmacological management

Pain is a common problem expressed by patients regardless of its stage and for its management we must take into account that it is a nociceptive and neuropathic pain. Nociceptive resulting from the activation of signaling molecules by tissue damage generated by inflammation and neuropathic by peripheral neuroplastic changes and central sensibilization generated by chronic inflammation [23,24]. For the management of acute pain, it is recommended to use paracetamol, topical or systemic non-steroidal anti-inflammatory drugs (NSAIDs), the use of opioids is avoided due to the chronicity of the disease and the risk of dependence, so short cycles will be used only if the pain is refractory. Regarding chronic pain, the use of NSAIDs is recommended as a first line and as a second line there are pregabalin, gabapentin, selective serotonin reuptake inhibitors and serotonin-norepinephrine reuptake inhibitors [[25], [26], [27], [28], [29], [30], [31]].

The management of the HS will be carried out depending on the Hurley classification **(**Table 4**)**; Thus, for stage I, topical clindamycin 1% can be used, it has been shown to reduce pustules but has no effect on inflammatory nodules or abscesses [28], so the mainstay is management with systemic antibiotics with tetracyclines, being of choice doxycycline at a dose of 100 mg every 12 h. Topical clindamycin (0.1%) twice daily was found to be as effective as oral tetracyclines (500 mg) for stage I lesions [4]. In the second line for stage I but first line for stage II, rifampicin associated with clindamycin, both at doses of 300 mg twice a day for 10 days, with a response range of 71–85% but with the risk of infection by *Clostridium difficile* [[31], [32], [33], [34]]. As a third line, mainly as rescue therapy for patients with Hurley stage III, ertapenem 1 g a day for 6 weeks, with a highly effective but a high level of relapses after it discontinuation. Dapsone is considered relatively ineffective because the response is approximately 38% and it is not effective in Hurley stage 3, the dose used was 50 mg per day at the beginning with a progressive increase until 200 mg per day for at least 3 months. It is important to note that improvement with antibiotic management has also been associated with its anti-inflammatory properties, as seen in tetracyclines that suppress lymphocytes, neutrophils, and histiocytes [[31], [32], [33], [34]].

**Table 4:** Treatment HS [4,40].

Treatment	Type of medicine	Dose	Indications	Adverse reactions
Topical Clindamicyn	Lincosamide	0,1% (Topical) 12 h (6-8 weeks)	Stage I injuries	Cutaneous xerosis, paraesthesia, burning pain, irritation.
Clindamicyn	Lincosamide	300 mg (OR) 12 h (10 days)	Stage I, II, III, injuries.	Dispepsia, metallic taste, nausea, emesis.
Rifampicin	Rifamycins	600 mg (OR) day (12 weeks) + clindamicyn.	Stage I, II, III, injuries.	Nausea, yellow discoloration of skin and teeth, drowsiness.
Ertapenem	Carbapenem	1gr (IV) day (6 weeks)	Stage I, II, III, injuries.	Thrombophlebitis, Thrombocytosis.
Dapsone	Sulfonamide	50 mg–200 mg (OR) day (3 weeks)	Stage I, II, injuries.	Insomnia, nausea, emesis, hemolysis.
Minocycline/colchicine	Tetracycline/anti-inflamatory	100 mg/0,5 mg (OR) 12 h.	Stage I, III, injuries.	Diarrhea, genitourinary itching.
Doxycycline	Tetracycline	100 mg (OR) 12 h.	Stage I, II, injuries.	Pruritus, lack of apetite, nausea, emesis.
Acitretin	retinoid	0,25–0,88 mg/kg (3-12 weeks)	Stage II, III, injuries.	Xerosis, sunburn, peeling.
Infliximab	(TNF-alpha).	7,5-10 mg/kg (IV) 4 weeks.	Stage II, III, injuries.	Dyspepsia, rhinorreha, nausea, emesis.
Adalimumab	(TNF-alpha).	40–80 mg (SC) 24 weeks	Stage II, III, injuries.	Enrythema, bruise, nausea, emesis.
Prednisolone	Corticoesteroid	10 mg (OR) day (4-6 weeks)	Stage I, II, injuries.	Weak skin, infections, nausea.

*OR: Oral route, **IV**: Intravenous, **SC**: Subcutaneous.

It's important to mention to the use of hormonal therapy that includes contraceptives and antiandrogens such as spironolactone and cyproterone, particularly in females, due to the onset of the disease at puberty, improvement after menopause, and fluctuations in disease severity during pregnancy and menses [35,36]. Studies have shown that the use of minocycline at a dose of 100 mg in conjunction with colchicine at a dose of 0.5 mg twice a day have shown satisfactory results. [4]; Acitretin a second-generation oral retinoid which has descriptions of response to treatment of acute lesions doses of 0.25–0.88 mg/kg (3-12 months). Currently research is being carried out with inflammatory mediators such as inhibitors of different interleukins such as IL-1, IL-17, IL-12, IL-23, for which there are still studies that support or completely reject this practice; TNF inhibitors such as infliximab are also found with a recommended dose of 7.5 mg/kg every 4 weeks increasing the dose to 10 mg/kg every 4 weeks if necessary [[37], [38], [39]]. The Adalimumab dose of 80 mg for the first two weeks and subsequently 40 mg for 24 weeks, there was no increase in the number of abscesses and fistulas caused by HS [40]. Corticosteroids are another option that can be considered, which can be used systemically or locally, a retrospective study was carried out with 13 patients using prednisolone 10 mg/day where a significant improvement was seen at 4–6 weeks and in some patients the remission of the lesions [41].

## 6 Surgical management

Due to the deterioration of the quality of life, patients are motivated to improve their current state through surgery. Surgical management encompasses various approaches, from the simplest such as incision and drainage that alleviate symptoms to more complex ones that require larger resections such as complete excision with curative intentions [42,43]. Although the use of these therapies is recommended from the initial stages of diagnosed HS, this does not always happen due to late diagnosis of the disease. A combined treatment of surgery plus pharmacological management (immunosuppressive and bacterial treatment) is recommended to reduce inflammation and complications [44], and the use of images is recommended to evaluate and confirm the depth and limits of the lesions.

*Incision and drainage*: Indicated for early stages (Hurley I). This therapy is not curative, it has a high recurrence rate, but it is recommended to relieve the pain of an acute abscess [45,46].

*Desroofing*: Consists of dissecting the upper wall or the roof of the fistulous tract or the persistent nodule, depending on the Hurley (I or II). This management reports a lower rate of recurrence [47,48].

*Laser therapy*: Two main methods of laser therapy are carbon dioxide excision, which has shown 71% and 99% cure, with 29% recurrence [[45], [46], [47], [48], [49], [50], [51]]. The second method is long-pulsed Nd:YAG laser excision, in which pilosebaceous cells are destroyed, studies showed that 90% of patients had a 72% reduction in HS lesions [52,53]. A study of four patients showed a 100% cure rate. A study of four patients who were treated with both laser therapies showed a 100% cure rate [[54], [55]].

*STEEP (Skin-tissue sparing incision with electrosurgical peeling)*: Saves healthy subcutaneous tissue and eliminates injured and fibrotic tissue that, if preserved, can generate recurrences. This favors the cosmetic result while generating less contractures in the skin and also requires less time of recovery [40,56].

*Complications*: Hemorrhage, infection, hypergranulation, flap necrosis, axillary plexus or vessel injury, venous thrombosis, hypertrophic and keloid scars.

## 7 Final considerations

HS is a disease characterized by a chronic inflammatory process at the level of the skin and that manifests with painful nodules, abscesses and fistulas that are difficult to diagnose due to their similarity to other pathologies, few data reported in the literature and limitations of epidemiological characteristics [56].

It is important to remember the location of HS, especially at the level of the folds such as axillary, inguinal, perianal, inframammary and femoral regions, where the apocrine sweat glands are located. At the beginning it presents with hyperplastic changes in the follicular epithelium, alteration of the microbiome in the skin and keratinocyte hyperplasia, thus determining a vicious cycle of chronic inflammation with severe and chronic outcomes, among which are lymphedema, scrotal elephantiasis, interstitial keratitis and even associated with squamous cell carcinoma in areas with chronic scars. Finally, the disease interferes with quality of life and difficult interpersonal relationships, with great psychological repercussions [57].

This study has some limitations. First of all, the published evidence regarding prevalence and incidence, diagnostic and therapeutic techniques, as well as advances in general management by general practitioners, comes almost exclusively from high-income countries. Therefore, this information cannot be extrapolated to all regions of the world, due to the lack of infrastructure and technology, as well as the availability of specialized services. Second, although this review is focused on providing information for the suspicion and diagnosis of hidradenitis suppurativa by general practitioners, the available data and recommendations are primarily intended for dermatology specialists, so there may be a gap in knowledge regarding the understanding of hidradenitis suppurativa. Finally, due to the lack of evidence, it was not possible to perform an analysis of the real problem and possible solutions in the context of low- and middle-income countries, where such population also presents a large number of risk factors such as obesity, low socioeconomic status, smoking and exposure to areas that favor the reproduction of the biological cycle of abundant microorganisms, as well as pollution. In addition, no evidence was found regarding molecular studies on the pathophysiology and severity or complication of this condition, therefore, this topic was also not discussed.

To date and after the review of the literature for a period of 3 years, search literature and systematics reviews, surgical resection of the lesions continues to be the cornerstone of the treatment and despite multiple drugs used based on the pathophysiology of this pathology, such as antibiotics, retinoids and biological treatments, it is still poorly specified their efficacies. It is necessary to inquire about the effectiveness with which general practitioners manage hidradenitis suppurativa, in order to specifically identify strengths and weaknesses, since these professionals constitute the first line of health care. Also, it is imperative to develop research that investigates genetic, clinical and sociodemographic factors on the genesis and progression of HS in low- and middle-income countries. In this way, it would be possible to synthesize the evidence and carry out studies with the highest level of evidence, in order to be able to establish solid recommendations on diagnosis, management, rehabilitation and quality of life in all regions of the world, with the aim of reducing the time of diagnosis and reducing the associated complications. Considering that the definitive approach is aggressive (surgical resection), everything possible must be done to prevent this condition from reaching this stage.

## 8 Conclusions

HS is a rare chronic inflammatory skin disease, that is until today not well known, the causes, pathophysiology, specificity of clinical manifestations and diagnostic methods are nonspecific and the diagnosis its late, development severe complications. It is necessary to conduct more research and create a clinical guide for the diagnosis and management of HS, generating answers to what we do not know.

## Declaration of competing interest

None.

## Sources of funding

None.

## Ethical approval

It is not necessary.

## Trial registry number

1. Name of the registry: Not applicable.
2. Unique Identifying number or registration ID: Not applicable.
3. Hyperlink to your specific registration (must be publicly accessible and will be checked): Not applicable.

## Author contribution

All authors equally contributed to the analysis and writing of the manuscript.

## Guarantor

Sabrina Rahman. Department of Public Health, Independent University-Bangladesh, Dhaka, Bangladesh. sabrinaemz25@gmail.com.

## Provenance and peer review

Not commissioned, externally peer reviewed.
